# A novel prognosis marker based on combined preoperative carcinoembryonic antigen and systemic inflammatory response for resectable gastric cancer

**DOI:** 10.7150/jca.52299

**Published:** 2021-01-01

**Authors:** Yubin Ma, Junpeng Lin, Jianxian Lin, Junfang Hou, Qin Xiao, Fang Yu, Zhijun Ma, Ping Li, Ruhong Tu, Jianwei Xie, Chaohui Zheng, Su Yan, Changming Huang

**Affiliations:** 1 Department of Gastrointestinal Oncology, Qinghai University Affiliated Hospital, Xining, People's Republic of China.; 2 Department of Gastric Surgery, Fujian Medical University Union Hospital, Fuzhou, People's Republic of China.

**Keywords:** gastric cancer, carcinoembryonic antigen, systemic inflammatory response, nomogram, prognosis

## Abstract

**Background:** Carcinoembryonic antigen (CEA) is one of the important indexes for the diagnosis and prognosis of gastrointestinal cancer. Systemic inflammatory response (SIR) is closely related to the occurrence and development of gastrointestinal cancer.

**Methods:** A total of 803 patients who underwent radical gastrectomy in Qinghai University Affiliated Hospital from January 2012 to December 2016 were included as training set. Multivariable Cox proportional hazard regression was used to identify associations with outcome of gastric cancer (GC). CNLR was established by combining CEA and the neutrophils to lymphocytes ratio (NLR, a typical parameter in SIR) to generate a novel prognostic score system and its prognostic value was externally validated.

**Results:** Multivariate analysis showed that CEA and NLR were independent prognostic factors for GC patients (both *p* < 0.05). A higher CNLR was significantly associated with older age, male sex, larger tumor size, vascular invasion and advanced stages (all *p* < 0.05). Patients with higher CNLR had poor prognosis than those with lower CNLR (*p* < 0.05). Multivariate analysis showed that CNLR was an independent prognostic factor (*p* < 0.05). Incorporation of the CNLR into a prognostic model including age and TNM stage generated a nomogram, which predicted accurately 3- and 5-year survival for GC patients. And similar results were obtained in the external validation set.

**Conclusions:** The CNLR prognostic scoring system established by combining CEA and NLR is an independent prognostic factor for GC, which can be incorporated into the traditional TNM staging to improve the prediction of long-term survival outcomes.

## Introduction

Gastric cancer (GC) is the fifth most common cancer in the world and the third leading cause of cancer-related death 1. To date, although great progress has been made in the treatment of GC, the long-term prognosis of patients with advanced GC remains poor. Therefore, how to identify high-risk GC patients early and provide more active comprehensive treatment will play a key role in the treatment effect as well as follow-up treatment evaluation.

In clinical practice, the detection of serum tumor marker (STM) is an ideal choice in terms of cost, convenience, and noninvasiveness. Carcinoembryonic antigen (CEA) is a STM that has been widely used in the diagnosis and monitoring of gastrointestinal cancer for a long time. CEA is closely related to the prognosis of GC 2-4. Increasing evidence has demonstrated that systemic inflammatory response (SIR) plays an important role in the occurrence and development of cancer 5, 6. Many parameters of SIR, such as the neutrophil to lymphocyte ratio (NLR), the platelet to lymphocyte ratio (PLR), and the lymphocyte to monocyte ratio (LMR), are closely related to the prognosis of various tumors 7. However, previous studies have often assessed the influence of a single factor on the long-term prognosis of patients. This study aims to screen the STM and SIR indicators that affect the prognosis of patients through multivariate analysis and to evaluate the effect of these indicators on the prognosis of patients with GC to further improve the predictivity accuracy of long-term prognosis evaluation based on TNM staging.

## Materials and methods

### General information

The clinicopathological data of patients diagnosed with primary gastric adenocarcinoma in Qinghai University Affiliated Hospital from January 2012 to December 2016 and treated with radical surgery (training set) were used for retrospective analysis. The inclusion criteria of the patients were as follows: (1) histopathology was consistent with gastric adenocarcinoma; (2) anti-inflammatory drugs were not used within 1 week before the operation; (3) no trauma, renal failure, and/or liver failure and infectious diseases; and (4) serum CEA and blood routine test were performed within 1 week before the operation. The exclusion criteria were as follows: (1) T4b (tumor invades adjacent structures/organs could not be resected by R0); (2) peritoneal spread or distant organ metastasis was confirmed during or after the operation; (3) neoadjuvant chemotherapy before the operation; and (4) incomplete clinicopathological data. Additional external validation was performed using a dataset from Fujian Medical University Union Hospital between January 2012 to December 2013, which satisfied the aforementioned inclusion and exclusion criteria. Finally, a total of 803 cases in the training set and 794 cases in the validation set were included in this study. According to Japan's 14th edition of the regulations for the treatment of GC, the scope of gastrectomy was selected, and the lymph nodes around the stomach were cleaned 8. TNM staging was carried out according to the 8th edition of the American Joint Committee on Cancer (AJCC) staging standard published in 2016 9.

### Inflammation index and the definition of CEA

One week before the operation, blood samples were collected to count and classify the blood cells using semiconductor laser flow cytometry 10, 11, detect albumin (ALB) with the bromocresol green method 12, and detect tumor markers with chemiluminescent immunoassay 13, 14 to obtain neutrophils, platelets, lymphocytes, monocytes, hemoglobin, serum ALB, and serum CEA. NLR was calculated by dividing the absolute value of neutrophils by the absolute value of lymphocytes. PLR was calculated by dividing the absolute value of platelets by the absolute value of lymphocytes. LMR was calculated by dividing the absolute value of lymphocytes by the absolute value of monocytes. The optimal cut-off values of NLR, PLR, LMR, Hb, ALB, and CEA were calculated using X-tile software 15 (http://www.tissuearray.org/rimmlab/), and the values were 1.86, 135.87, 4.75, 109 g/L, 38.00 g/L, and 3.04 ng/mL, respectively.

### Postoperative follow-up

All patients were followed up every three months in the first two years and every six months in the following three to five years. The last follow-up was conducted in April 2019. Routine follow-up examination items included physical examination, laboratory examination (blood routine examination, blood biochemistry, and tumor markers), chest X-ray, abdominal color ultrasound, chest and abdomen CT, and gastroscopy once a year. Overall survival (OS) was defined as the time from the beginning of surgery to the last follow-up, the time of death caused by any reason, or the time of the end of follow-up in the database (such as loss to follow-up visit and death from other diseases).

### Statistical methods

All data were processed by SPSS 18.0. The distribution of inflammatory indexes, CEA, and other clinicopathological data was summarised using descriptive statistical methods. Continuous data were compared across cohorts using the unpaired t test and categorical data were compared using the chi-square test or the Fisher exact test where appropriate. The Kaplan-Meier method was used to calculate the survival rate, and survival curves were compared using the log-rank test. Univariable and multivariable Cox proportional hazard regression were used to identify independent prognostic factors. A nomogram was created with R software (version 3.5.1) using 'rms' package. Calibration plots were generated to examine the performance characteristics of the predictive nomogram. The predictive accuracy of the models was evaluated using the Harrell's Concordance index (C-index) and time-dependent receiver-operating characteristic (ROC) curve. The R package “timeROC” was used for time-dependent ROC curve analyses. The difference was considered statistically significant when *p* < 0.05.

## Results

### Patient characteristics

A total of 803 cases were included in the training set, including 606 males (75.5%) and 197 females (24.5%). The median age was 58 years (interquartile range [IQR]: 49-64 years). A total of 262 cases were classified as stage I, representing 32.6% of the cases. In addition, 210 cases were in stage II, representing 26.2% of the cases. In total, 331 cases were in stage III, representing 41.2% of the cases. The median CEA level was 2.13 ng/ml (IQR: 1.38-3.56), and the median NLR was 2.01 (IQR: 1.45-3.49). A total of 794 cases were included in the validation set, including 604 males (76.1%) and 190 females (23.9%). The median age was 61 years (IQR: 55-68 years). A total of 221 cases were classified as stage I, representing 27.8% of the cases. In total, 194 cases were in stage II, representing 24.4% of the cases. Moreover, 379 cases were in stage III, representing 47.9% of the cases. The median CEA level was 2.60 ng/ml (IQR: 1.60-4.60), and the median NLR was 2.11 (IQR: 1.57-2.88) (Supplementary Table 1).

### Survival analysis

The median follow-up time was 42 months (range, 1-106 months), and the overall 5-year survival rate was 66.6% in the training set. In the validation set, the corresponding values were 57 months (range, 1-77 months) and 67.5%, respectively. Univariate analysis showed that CEA and SIR, including NLR, PLR and LMR, were closely related to the prognosis in the training set (all *p* < 0.05, Table 1), and the other relevant clinicopathological data included age, tumor size, differentiation degree, vascular invasion, pTNM stage, and ALB content (all *p* < 0.05, Table 1). However, multivariate analysis showed that CEA (hazard ratio [HR]: 1.439, 95% CI: 1.125-1.839, *p* = 0.004) and NLR (HR: 1.297, 95% CI: 1.007-1.672, *p* = 0.044, Table 1) were independent prognostic factors for GC.

### Establishment of a combined index based on CEA and LMR: CNLR

**Figure 1:**
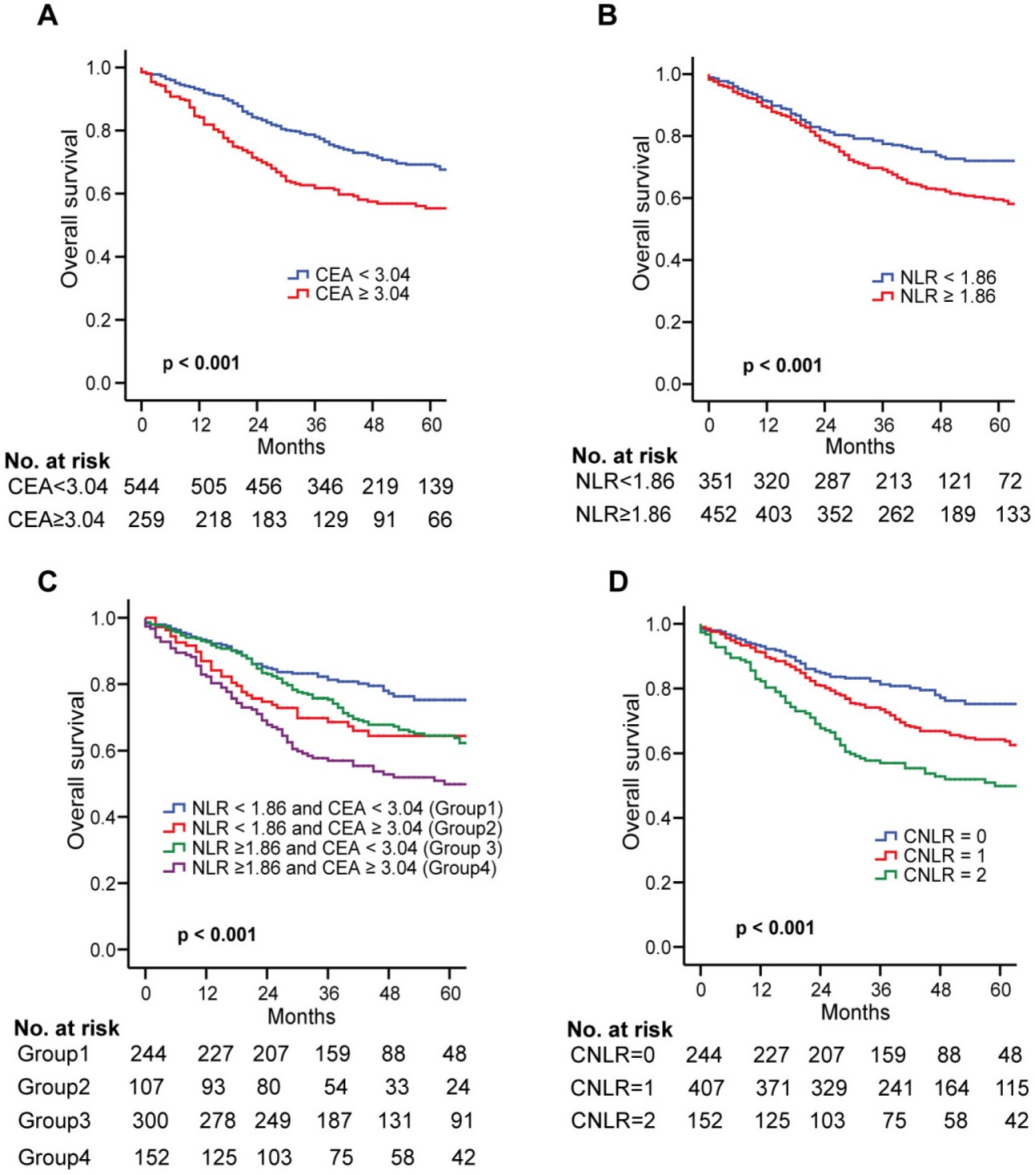
Kaplan-Meier survival curves for OS in patients undergoing GC radical resection according to preoperative NLR and CEA in the training set. Kaplan-Meier analysis for OS according to **a** preoperative CEA; **b** preoperative NLR; **c** combination of preoperative NLR and CEA; and **d** CNLR. *Abbreviations: OS* overall survival, *GC* gastric cancer, *NLR* neutrophils to lymphocytes ratio, *CEA* carcinoembryonic antigen.

In the training set, the Kaplan-Meier survival curve showed that the high CEA (≥ 3.04) and NLR (≥ 1.86) were closely related to the poor prognosis of GC (both *p* < 0.001, Fig. 1a and b). All the patients were divided into four groups by combining CEA and NLR. Significant differences exist among the four subgroups (Fig. 1c). However, in subgroups of either high CEA or high NLR, the OS was quite similar (*p* > 0.05, Fig. 1c). Therefore, we combined the two groups to establish the CNLR defined as follows: preoperative low CEA (< 3.04) with low NLR (< 1.86) was given a score of 0; preoperative high CEA (≥ 3.04) with low NLR (< 1.86) or preoperative low CEA (< 3.04) with high NLR (≥ 1.86) was given a score of 1; preoperative high CEA (≥ 3.04) with high NLR (≥ 1.86) was given a score of 2 (Supplementary Table 3).

In the training set, there were 244 cases (30.4%) with a CNLR score of 0, 407 cases (50.7%) with a CNLR score of 1, and 152 cases (18.9%) with a CNLR score of 2. The relationship between CNLR and clinicopathological data revealed that a higher CNLR was significantly associated with older age, male sex, larger tumor size, vascular invasion and advanced stages (all *p* < 0.05, Table 2), but not associated with ASA score, tumor location, tumour differentiation, adjuvant chemotherapy, and morbidity (all *p* > 0.05, Table 2). In the validation set, there were 197 cases (24.8%) with a CNLR score of 0, 369 cases (46.5%) with a CNLR score of 1, and 228 cases (28.7%) with a CNLR score of 2. The CNLR score was also associated with age, gender, tumor location, tumor size, vascular invasion, and pTNM stage (*p* < 0.05, Supplementary Table 2).

### Correlations of the CNLR score with OS

The Kaplan Meier survival curve showed that an increased CNLR significantly associated with poor prognosis of GC (CNLR score 0 vs CNLR score 1, *p* = 0.002; CNLR score 0 vs CNLR score 2, *p* < 0.001; CNLR score 1 vs CNLR score 2, *p* = 0.001, Fig. 1d). The prognostic accuracies of the CNLR and each of its components-CEA and NLR, were compared by using AUCs for the prediction of 5-year OS. The AUCs for the CNLR, CEA and the NLR were 0.607 (95% confidence interval [CI] 0.572-0.641), 0.573 (95% CI 0.538-0.607) and 0.569 (95% CI 0.534-0.604), respectively. According to the Z test method, the AUC for the CNLR was significantly higher than that for CEA and the NLR (both *p* < 0.05).

Multivariate Cox regression analysis showed that CNLR was an independent prognostic factor for patients with GC (CNLR = 1: HR = 1.397, *p* = 0.040; CNLR = 2: HR = 1.844, *p* < 0.001). The other independent risk factors included age and TNM stage (*p* < 0.001). In the validation set, multivariate Cox regression analysis also showed that CNLR was an independent prognostic factor for patients with GC (*p* = 0.004) (Table 3).

### Establishment of a nomogram based on CNLR

**Figure 2:**
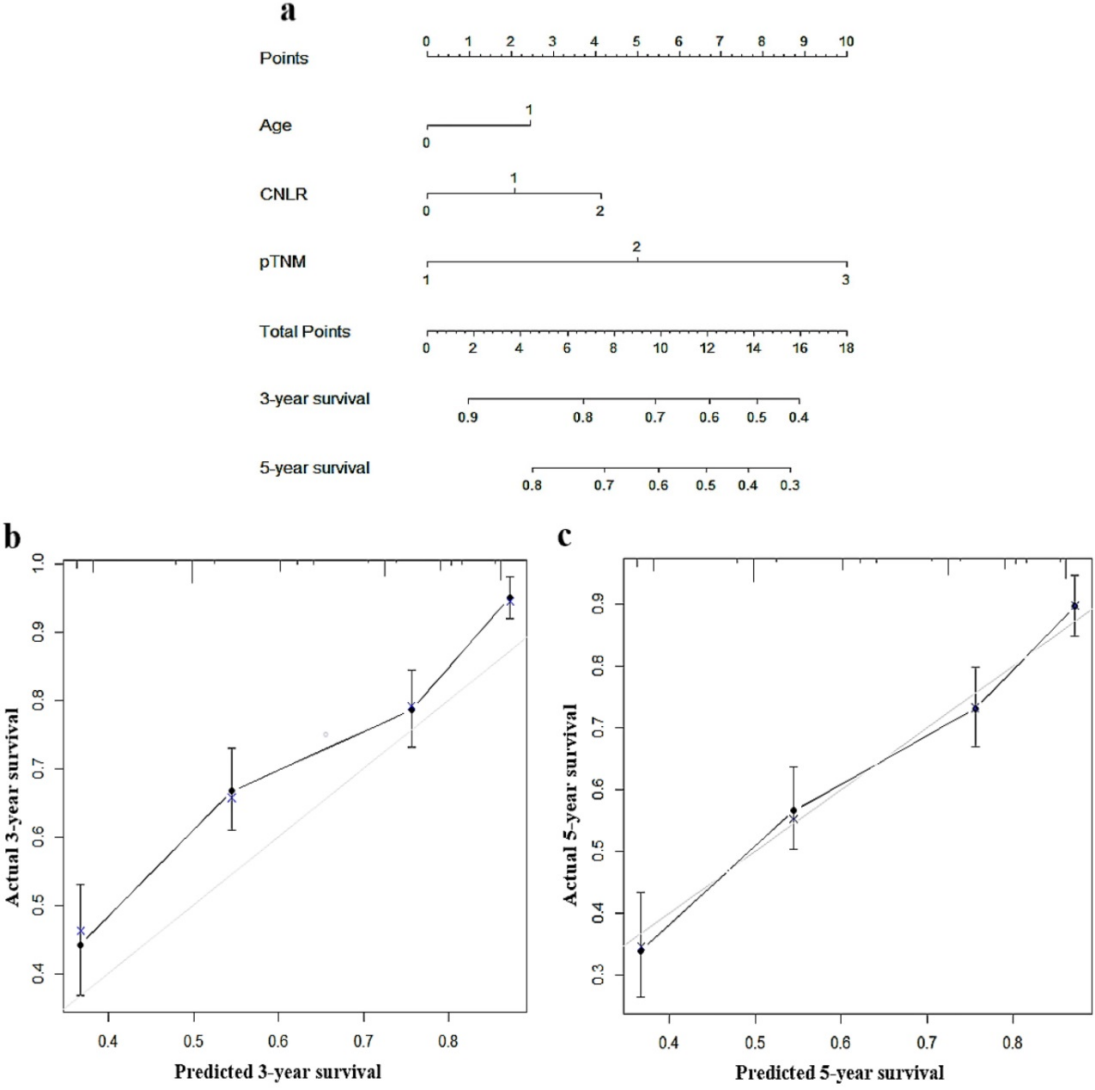
Nomogram for predicting 3-year and 5-year survival of GC patients after surgery in the training set **a**, calibration plot of the nomogram for 3-year survival **b** and 5-year survival **c.** *Abbreviations:* GC gastric cancer.

**Figure 3:**
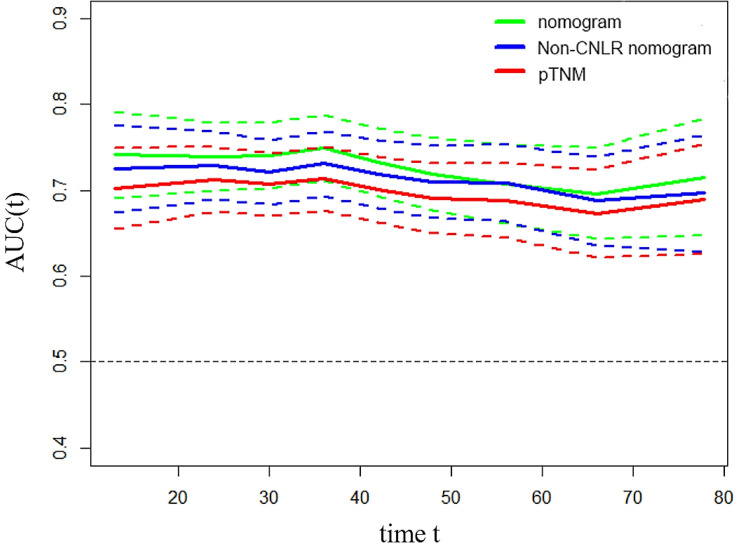
Time-dependent ROC curves for the nomogram, non-CNLR nomogram, and pTNM for the prediction of OS in the training set. The horizontal axis represents the years after surgery, and the vertical axis represents the estimated area under the ROC curve for survival at the time of interest. Green, blue, and red solid lines represent the estimated AUCs of the nomogram, non-CNLR nomogram, and pTNM, respectively. Broken lines represent the 95% confidence interval of each AUC. *Abbreviations: ROC* receiver-operating characteristic, *CNLR* combination of carcinoembryonic antigen and neutrophils to lymphocytes ratio, *OS* overall survival, *AUC* area under the curve.

To predict the prognosis of GC quantitatively and accurately, we established a nomogram (Fig. 2a) based on the independent prognostic factors, including CNLR, pTNM, stage, and age. The results showed that the nomogram could more accurately predict the overall 3-year and 5-year survival of GC after the operation in the training set (Fig. 2b and c). The C-index of the nomogram based on CNLR was 0.704 (95% CI, 0.675-0.733), which was significantly increased compared with the C-index of the non-CNLR nomogram (0.691; 95% CI, 0.662-0.721; *p* = 0.020) and that of the pTNM stage prognosis model (0.677; 95% CI 0.649-0.705; *p* < 0.001). In addition, we also compared the predictive value of the three prognostic models for the prognosis of GC by establishing a time-dependent ROC curve (Fig. 3). The results showed that the nomogram based on CNLR was superior to the non-CNLR nomogram and the pTNM stage model during the follow-up period, indicating that the prediction efficiency of the nomogram based on CNLR was greater than that of the non-CNLR nomogram and the pTNM stage prognosis model.

Similar results were obtained by establishing a nomogram for the validation set. The C-indexes of the three prognostic models were 0.750 (95% CI, 0.722-0.780), 0.740 (95% CI, 0.712-0.670, *p* = 0.163), and 0.719 (95% CI, 0.693-0.745, *p* < 0.001) (Supplementary Fig. 2a, b, and c). The time-dependent ROC curve showed that the CNLR-based nomogram performed better than the non-CNLR nomogram and pTNM staging in prognosis evaluation of GC (Supplementary Fig. 3).

## Discussion

At present, the TNM staging system established by the AJCC and Union for International Cancer Control is the most commonly used tumor staging standard in the world and plays an extremely important role in the evaluation of treatment effect and patient prognosis 9. However, tumor heterogeneity is common among different patients suffering from the same type of malignant tumor, which often causes difficulties in the treatment and prognosis evaluation of malignant tumors. In some studies, it has been reported that STM and SIR before treatment can independently predict the prognosis of patients with GC 16, 17. However, these studies have typically evaluated one single indicator based on a relatively small sample group. Based on a variety of large data, this study evaluated the impact of STM and SIR and their combined effects on the prognosis of patients with GC undergoing surgery. In this study, we found that CEA and NLR were independent prognostic factors for the prognosis of patients with GC.

STM plays an important role in the diagnosis and prognosis of tumors, and among them, CEA detection is the most widely used and most accessible auxiliary diagnosis method for the prognosis of gastrointestinal cancer 18. Studies have demonstrated that the transcription and secretion of CEA are regulated by the Smad3-mediated tumor growth factor and transforming growth factor β (TGF-β) signaling pathway, and TGF-β plays an important role in cell proliferation and differentiation, embryonic development, extracellular matrix formation, immune regulation, and tumor development 19. In addition, the TGF-β signaling pathway regulates Smad3 and LPS/TLR4 signal transduction, activates NF-KB and other nuclear transcription factors, and generates a large number of inflammatory mediators, leading to SIR 20, 21. In addition, *in vitro* experiments have demonstrated that the CEA receptor on the surface of macrophages after differentiation induction can combine with CEA and induce macrophages to rapidly secrete a large amount of tumor necrosis factor (TNFa), interleukin-1 (IL-l), interleukin-6 (IL-6), interleukin-10, and other cytokines, which affect tumor immunity via multiple mechanisms 22. These results show that CEA affects the growth and apoptosis of cancer cells through complex signaling pathways in the host, which has a good prognostic value, and it is recommended by the American Society of Clinical Oncology and the European Society for Medical Oncology as the gold standard for follow-up of gastrointestinal cancer patients 23, 24. However, the sensitivity and specificity of CEA alone for the diagnosis and prognosis of GC is low 3, 25. In our study, the CEA positive rate of patients with GC was only 32.3%.

The correlation between SIR and malignant tumors has become a hot research topic at this stage. Research demonstrates that inflammatory factors are closely related to the occurrence and progress of malignant tumors, which can cause the invasion and metastasis of malignant tumors 5, 26, 27. Related studies have revealed that a series of inflammatory cells and innate immune system signaling molecules, such as neutrophils, lymphocytes, platelets, and monocytes, are involved in tumor progression 28. In this study, we selected LMR, PLR, and NLR, which are commonly used as indicators of SIR for analysis. The results showed that only preoperative NLR was an independent risk factor affecting the prognosis of patients with GC. NLR is an important indicator of the inflammatory response. On one hand, neutrophils generate and release a large amount of VEGF-A, IL-l, IL-6, IL-8, TNFa, TGF, and other inflammatory factors and cytokines 20 through the TGF-β signaling pathway in an inflammatory response, generating a microenvironment suitable for tumor survival and affecting tumor immunity to induce tumor angiogenesis, proliferation, and invasion 29-31. In addition, systemic inflammation significantly reduces the cellular immune capacity of the body by reducing the capacity of CD4^+^ T lymphocytes of the host and inhibiting the increase in CD8^+^ T lymphocytes 32, 33. Therefore, NLR is an important marker representing the balance between the tumor inflammatory pathway and the anti-tumor immune system function. The increase in NLR not only represents the increase in inflammatory cells and increasing tumor growth in the microenvironment but also represents the inhibition of lymphocyte-mediated immune response to promote the immune escape of tumor cells, which is closely related to tumor invasion, angiogenesis, and metastasis. As a result, the overall survival of patients with high NLR was poor 34. Therefore, compared with LMR and PLR, which represent SIR, preoperative high NLR had a greater impact on the prognosis of patients with GC, which is consistent with some previous research results 35-39.

Based on the above studies and reports, we found that CEA and NLR have a close internal relationship in the TGF-β signaling pathway, release of cytokines, reduction of the immune capacity, and other aspects. Their mutual influence and joint action lead to the occurrence and development of tumors and poor prognosis of patients. Therefore, in this study, CNLR, a new prognostic scoring system established by CEA and NLR, was identified as an independent prognostic factor for patients with GC, and the same results were obtained in the validation set. Therefore, as a combined index of CEA and NLR, CNLR can better reflect the comprehensive effect of CEA and NLR on tumor progression. In addition, this study established a new nomogram prognosis model by incorporating CNLR, pTNM, stage, and age. The correction curve showed that the nomogram could well predict the prognosis of patients with GC. The accuracy of the nomogram with CNLR in predicting the prognosis of GC was significantly better than that of the non-CNLR nomograms and pTNM. Similar results were also obtained in the validation set. Therefore, in clinical practice, CNLR can be used as a supplement to the traditional TNM staging method for preoperative risk stratification and prognostic evaluation of patients with GC to effectively guide the subsequent treatment strategy.

However, there are some limitations to this study. First, selection bias may exist due to the retrospective nature of the study. Second, some inevitable confounding factors may be involved in the study, such as smoking, drinking, and chronic inflammation, which may affect the values of CEA and NLR. Third, the state of the patients before the blood tests done before surgery cannot be guaranteed to be completely consistent. However, despite the limitations mentioned above, this study for the first time found that preoperative CEA and NLR are closely related to the prognosis of GC through bulk data analysis and external validation. We have established a new and simple prognostic scoring system, CNLR, by combining these two indicators. CNLR can effectively predict the prognosis of patients with GC, which can be considered as a supplement to the traditional staging system in clinical practice to improve the prognosis evaluation of GC patients and guide the follow-up individualised treatment plan.

## Supplementary Material

Supplementary figures and tables.

## Figures and Tables

**Table 1:** Univariate and multivariate analysis of clinicopathological variables in relation to overall survival in patients undergoing radical gastrectomy in the training set

Clinicopathological features	Univariate analysis		Multivariate analysis	
	HR (95% CI)	*p*	HR (95% CI)	*p*
**Age (years)**				
<65	Reference		Reference	
≥65	1.626 (1.252-2.113)	<0.001	1.438 (1.104-1.872)	0.007
**Gender**				
Female	Reference			
Male	1.065 (0.805-1.408)	0.660		
**Nationality**		0.702		
Han	Reference			
Hui	0.939 (0.605-1.458)	0.780		
Zang	0.876 (0.559-1.372)	0.563		
Other	0.751 (0.445-1.269)	0.285		
**Familial genetic history**				
Negative	Reference			
Positive	1.003 (0.721-1.394)	0.988		
**ASA score**		0.631		
1	Reference			
2	1.160 (0.855-1.573)	0.340		
3	1.065 (0.546-2.076)	0.854		
**Tumour location**		0.661		
Upper	Reference			
Middle	1.008 (0.751-1.354)	0.956		
Lower	0.972 (0.686-1.376)	0.872		
Mixed	2.050 (0.643-6.532)	0.225		
**Tumour size (cm)**				
<4.3	Reference		—	
≥4.3	1.846 (1.451-2.347)	<0.001	—	0.132
**Tumour differentiation**				
Differentiated	Reference		—	
Undifferentiated	1.305 (1.023-1.664)	0.032	—	0.162
Vascular invasion	Reference	<0.001		0.125
Negative	Reference		—	
Positive	2.287 (1.708-3.062)	<0.001	—	0.044
Unknown	0.694 (0.405-1.190)	0.185	—	0.806
**pTNM stage**		<0.001		<0.001
I	Reference		Reference	
II	2.282 (1.504-3.463)	<0.001	2.156 (1.420-3.275)	<0.001
III	5.089 (3.537-7.322)	<0.001	4.567 (3.163-6.595)	<0.001
**Adjuvant chemotherapy**				
No	Reference			
Yes	0.936 (0.736-1.190)	0.590		
**CEA (ng/mL)**				
<3.04	Reference		Reference	
≥3.04	1.749 (1.373-2.229)	<0.001	1.439 (1.125-1.839)	0.004
**LMR**				
<4.75	Reference			
≥4.75	0.768 (0.600-0.984)	0.037	—	0.795
**PLR**				
<135.87	Reference			
≥135.87	1.427 (1.123-1.814)	0.004	—	0.759
**NLR**				
<1.86	Reference		Reference	
≥1.86	1.508 (1.172-1.941)	0.001	1.297 (1.007-1.672)	0.044
**ALB (g/L)**				
<38	Reference		—	
≥38	0.777 (0.607-0.994)	0.045	—	0.258
**Hb (g/L)**				
<109	Reference	0.100		
≥109	0.769 (0.563-1.052)	0.100		

*HR* hazard ratio, *CI* confidence ratio, *ASA* American Society of Anesthesiologists, *CEA* carcinoembryonic antigen, *LMR* lymphocytes to monocytes, *PLR* platelets to lymphocytes, *NLR* neutrophils to lymphocytes ratio, *ALB* albumin, *Hb* haemoglobin.

**Table 2:** The relationship between the CNLR and clinicopathological characteristics in patients undergoing radical gastrectomy in the training set

Clinicopathological features	CNLR			*p*
	0	1	2	
**Case**	244 (30.4%)	407 (50.7%)	152 (18.9%)	
Age (years, 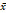 ± s)	55.02 ± 10.44	56.54 ± 9.99	58.63 ± 9.27	0.002
**Gender**				<0.001
Female	82 (33.6%)	89 (21.9%)	26 (17.1%)	
Male	162 (66.4%)	318 (78.1%)	126 (82.9%)	
**Nationality**				0.914
Han	182 (74.6%)	314 (77.1%)	113 (74.3%)	
Hui	22 (9.0%)	33 (8.1%)	12 (7.9%)	
Zang	24 (9.8%)	31 (7.6%)	16 (10.5)	
Other	16 (6.6%)	29 (7.1%))	11 (7.2%)	
**ASA score**				0.059
1	206 (84.4%)	311 (76.4%)	111 (73.0%)	
2	33 (13.5%)	79 (19.4%)	34 (22.4%)	
3	5 (2.1%)	17 (4.2%)	7 (4.6%)	
**Tumour location**				0.607
Upper	57 (23.4%)	99 (24.3%)	38 (25.0%)	
Middle	123 (50.4%)	216 (53.1%)	69 (45.4%)	
Lower	61 (25.0%)	89 (21.9%)	44 (28.9%)	
Mixed	3 (1.2%)	3 (0.7%)	1 (0.7%)	
Tumour size (cm, 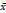 ± s)	3.82 ± 2.04	4.17 ± 2.09	4.67 ± 2.26	0.001
**Tumour differentiation**				0.498
Differentiated	114 (46.7%)	186 (45.7%)	78 (51.3%)	
Undifferentiated	130 (53.3%)	221 (54.3%)	74 (48.7%)	
**Vascular invasion**				0.001
Negative	91 (37.3%)	132 (32.4%)	41 (27.0%)	
Positive	108 (44.3%)	227(55.8%)	99 (65.1%)	
Unknown	45 (18.4%)	48 (11.8%)	12 (7.9%)	
**Adjuvant chemotherapy**				0.352
No	104 (42.6%)	183 (45.0%)	76 (50.0%)	
Yes	140 (54.7%)	224 (55.0%)	77 (50.0%)	
**pTNM stage**				<0.001
I	99 (40.6%)	132 (32.4%)	31 (20.4%)	
II	67 (27.5%)	107 (26.3%)	36 (23.7%)	
III	78 (32.0%)	168 (41.3%)	85 (55.9%)	
**Postoperative morbidity**				0.124
No	181 (74.2%)	326 (80.1%)	124 (81.6%)	
Yes	63 (25.8%)	81 (19.9%)	28 (18.4%)	

*CNLR* combination of carcinoembryonic antigen and neutrophils to lymphocytes ratio, *ASA* American Society of Anesthesiologists.

**Table 3:** Multivariate analysis of clinicopathologic variables in relation to overall survival in patients undergoing radical gastrectomy in the training and validation sets

Clinicopathological features	Multivariate analysis			
	Training set		Validation set	
	HR (95% CI)	*p*	HR (95% CI)	*p*
**Age**				
<65	Reference		Reference	
≥65	1.413 (1.084-1.841)	0.010	1.486 (1.135-1.945)	0.004
**pTNM stage**		<0.001		<0.001
I	Reference		Reference	
II	1.874 (1.185-2.964)	0.007	2.214 (1.137-4.313)	0.019
III	3.697 (2.402-5.690)	<0.001	9.883 (5.608-17.418)	<0.001
**CNLR**		<0.001		0.004
0	Reference		Reference	
1	1.397 (1.016-1.921)	0.040	1.038 (0.703-1.533)	0.851
2	1.844 (1.289-2.637)	0.001	1.646 (1.110-2.439)	0.013

*HR* hazard ratio, *CI* confidence interval, *CNLR* combination of carcinoembryonic antigen and neutrophils to lymphocytes ratio.

## References

[1] Kim ST, Cristescu R, Bass AJ, Kim KM, Odegaard JI, Kim K (2018). Comprehensive molecular characterization of clinical responses to PD-1 inhibition in metastatic gastric cancer. Nat Med.

[2] Marrelli D, Pinto E, De Stefano A, de Manzoni G, Farnetani M, Garosi L (2001). Preoperative positivity of serum tumor markers is a strong predictor of hematogenous recurrence of gastric cancer. J Surg Oncol.

[3] Nam DH, Lee YK, Park JC, Lee H, Shin SK, Lee SK (2013). Prognostic value of early postoperative tumor marker response in gastric cancer. Ann Surg Oncol.

[4] Wang W, Seeruttun SR, Fang C, Chen J, Li Y, Liu Z (2016). Prognostic significance of carcinoembryonic antigen staining in cancer tissues of gastric cancer patients. Ann Surg Oncol.

[5] Mantovani A, Allavena P, Sica A, Balkwill F (2008). Cancer-related inflammation. Nature.

[6] Elinav E, Nowarski R, Thaiss CA, Hu B, Jin C, Flavell RA (2013). Inflammation-induced cancer: crosstalk between tumours, immune cells and microorganisms. Nat Rev Cancer.

[7] Kao SC, Pavlakis N, Harvie R, Vardy JL, Boyer MJ, van Zandwijk N (2010). High blood neutrophil-to-lymphocyte ratio is an indicator of poor prognosis in malignant mesothelioma patients undergoing systemic therapy. Clin Cancer Res.

[8] Japanese Gastric Cancer Association (2011). Japanese gastric cancer treatment guidelines 2010 (ver. 3). Gastric Cancer.

[9] Amin MB (2016). AJCC Cancer Staging Manual. New York: Springer.

[10] National Committee for Clinical Laboratory Standards (2020). Reference leukocyte (WBC) differential count (proportional) and evaluation of instrumental methods, 2nd Edition. https://clsi.org/standards/products/hematology/documents/h20/.

[11] Barnes PW, McFadden SL, Machin SJ, Simson E (2005). The international consensus group for hematology review: suggested criteria for action following automated CBC and WBC differential analysis. Lab Hematol.

[12] Doumas BT, Peters T (2009). Origins of dye-binding methods for measuring serum albumin. Clin Chem.

[13] Zhao L, Sun L, Chu X (2009). Chemiluminescence immunoassay. Trends Anal Chem.

[14] Bi S, Yan Y, Yang X, Zhang S (2009). Gold nanolabels for new enhanced chemiluminescence immunoassay of alpha-fetoprotein based on magnetic beads. Chemistry.

[15] Camp RL, Dolled-Filhart M, Rimm DL (2004). X-tile: a new bio-informatics tool for biomarker assessment and outcome-based cut-point optimization. Clin Cancer Res.

[16] Ishigami S, Natsugoe S, Hokita S, Che X, Tokuda K, Nakajo A (2001). Clinical importance of preoperative carcinoembryonic antigen and carbohydrate antigen 19-9 levels in gastric cancer. J Clin Gastroenterol.

[17] Aurello P, Tierno SM, Berardi G, Tomassini F, Magistri P, D'Angelo F (2014). Value of preoperative inflammation-based prognostic scores in predicting overall survival and disease-free survival in patients with gastric cancer. Ann Surg Oncol.

[18] Gold P, Freedman SO (1995). Specific carcinoembryonic antigens of the human digestive system. J Exp Med.

[19] Han SU, Kwak TH, Her KH, Cho YH, Choi C, Lee HJ (2008). CEACAM5 and CEACAM6 are major target genes for Smad3-mediated TGF-β signaling. Oncogene.

[20] McCartney-Francis N, Jin W, Wahl SM (2004). Aberrant Toll receptor expression and endotoxin hypersensitivity in mice lacking a functional TGF-β1 signaling pathway. J Immunol.

[21] Kanamaru Y, Sumiyoshi K, Ushio H, Ogawa H, Okumura K, Nakao A (2005). Smad3 deficiency in mast cells provides efficient host protection against acute septic peritonitis. J Immunol.

[22] Aarons CB, Bajenova O, Thomas P, Andrews C, Heydrick S, Reed KL (2005). The response of differentiated THP-1 macrophages to carcinoembryonic antigen (CEA): a model system for Kupffer cell activation in metastatic colon cancer. Gastroenterology.

[23] Goldstein MJ, Mitchell EP (2005). Carcinoembryonic antigen in the staging and follow-up of patients with colorectal cancer. Cancer Investig.

[24] Schmoll HJ, Van Cutsem E, Stein AESMO, Valentini V, Glimelius B, Haustermans K (2012). Esmo consensus guidelines for management of patients with colon and rectal cancer. A personalized approach to clinical decision making. Ann Oncol.

[25] Wang W, Chen XL, Zhao SY, Xu YH, Zhang WH, Liu K (2013). Prognostic significance of preoperative serum CA125, CA19-9 and CEA in gastric carcinoma. Oncotarget.

[26] Balkwill F, Mantovani A (2001). Inflammation and cancer: back to Virchow?. Lancet.

[27] Wang H, Wang HS, Zhou BH, Li CL, Zhang F, Wang XF (2013). Epithelial-mesenchymal transition (EMT) induced by TNF-α requires AKT/GSK-3β-mediated stabilization of snail in colorectal cancer. PloS ONE.

[28] Cedrés S, Torrejon D, Martinez A, Martinez P, Navarro A, Zamora E (2012). Neutrophil to lymphocyte ratio (NLR) as an indicator of poor prognosis in stage IV non-small cell lung cancer. Clin Trans Oncol.

[29] Grivennikov SI, Greten FR, Karin M (2010). Immunity, inflammation, and cancer. Cell.

[30] Cools-Lartigue J, Spicer J, McDonald B, Gowing S, Chow S, Giannias B (2013). Neutrophil extracellular traps sequester circulating tumor cells and promote metastasis. J Clin Investig.

[31] Krzystek-Korpacka M, Matusiewicz M, Diakowska D, Grabowski K, Blachut K, Kustrzeba-Wojcicka I (2007). Impact of weight loss on circulating IL-1, IL-6, IL-8, TNF-alpha, VEGF-A, VEGF-C and midkine in gastroesophageal cancer patients. Clin Biochem.

[32] Menges T, Engel J, Welters I, Wagner RM, Little S, Ruwoldt R (1999). Changes in blood lymphocyte populations after multiple trauma: association with posttraumatic complications. Crit Care Med.

[33] Rabinowich H, Cohen R, Bruderman I, Steiner Z, Klajman A (1987). Functional analysis of mononuclear cells infiltrating into tumors: lysis of autologous human tumor cells by cultured infiltrating lymphocytes. Cancer Res.

[34] Kitamura T, Qian BZ, Pollard JW (2015). Immune cell promotion of metastasis. Nat Rev Immunol.

[35] Murthy BL, Thomson CS, Dodwell D, Shenoy H, Mikeljevic JS, Forman D (2007). Postoperative wound complications and systemic recurrence in breast cancer. Br J Cancer.

[36] Stotz M, Gerger A, Eisner F, Szkandera J, Loibner H, Ress AL (2013). Increased neutrophil-lymphocyte ratio is a poor prognostic factor in patients with primary operable and inoperable pancreatic cancer. Br J Cancer.

[37] Mano Y, Shirabe K, Yamashita YI, Harimoto N, Tsujita E, Takeishi K (2013). Preoperative neutrophil-to-lymphocyte ratio is a predictor of survival after hepatectomy for hepatocellular carcinoma: a retrospective analysis. Ann Surg.

[38] Shi P, Zhao Y, Deng X (2012). Clinical significance of preoperative neutrophil lymphocyte ratio (NLR) in predicting the prognosis of gastric cancer. J Mod Oncol.

[39] Shibutani M, Maeda K, Nagahara H, Noda E, Ohtani H, Sugano K (2013). Significance of preoperative neutrophil-to-lymphocyte ratio as a predictor of prognosis in patients with stage IV colorectal cancer. Cancer Chemother.

