# Supplementary material for: A novel prognosis marker based on combined preoperative carcinoembryonic antigen and systemic inflammatory response for resectable gastric cancer
Source: J Cancer. 2021 Jan 1;12(3):927–35. doi: 10.7150/jca.52299 (PMC7778548; doi:10.7150/jca.52299)
Supplement: Supplementary file 1 — Supplementary figures and tables. [file jcav12p0927s1.pdf]

Supplementary Fig. 1 Diagram representing the selection of the training set study population

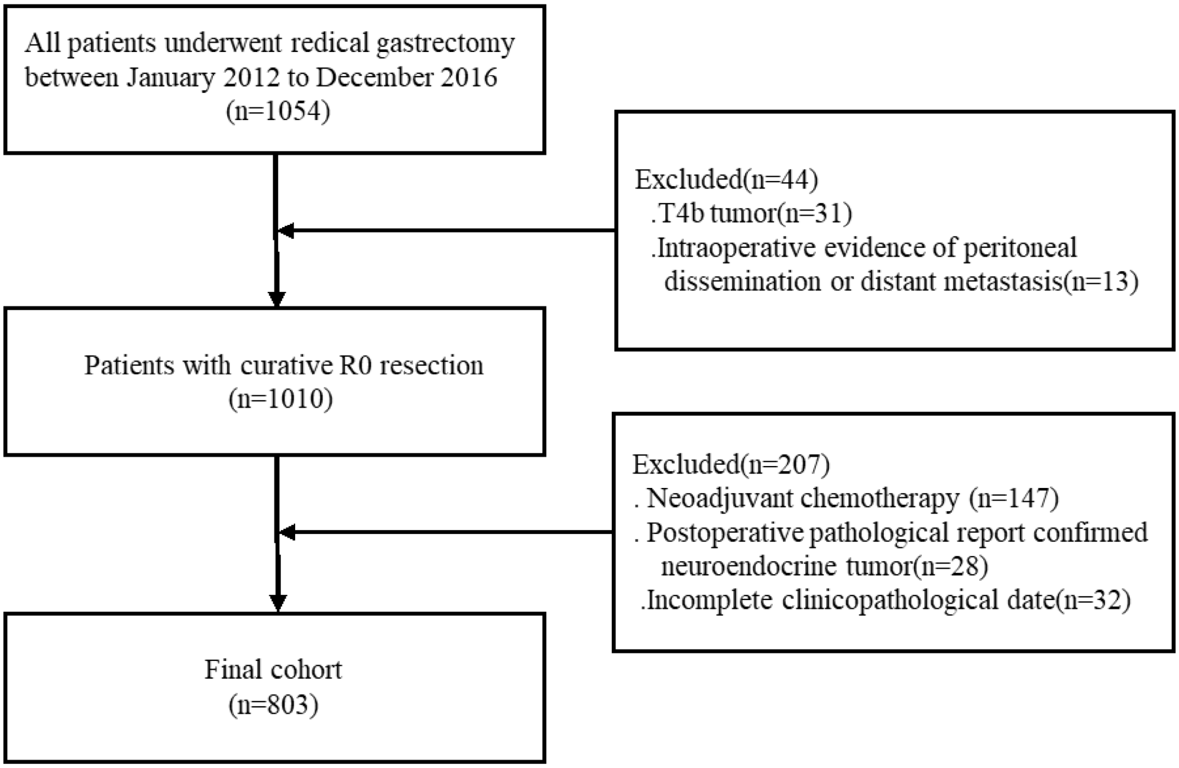

Supplementary Fig. 2 Nomogram for predicting 3-year and 5-year survival of GC patients after surgery in the validation set **a**, Calibration plot of the nomogram for 3-year survival **b** and 5-year survival **c**

Abbreviations: GC gastric cancer

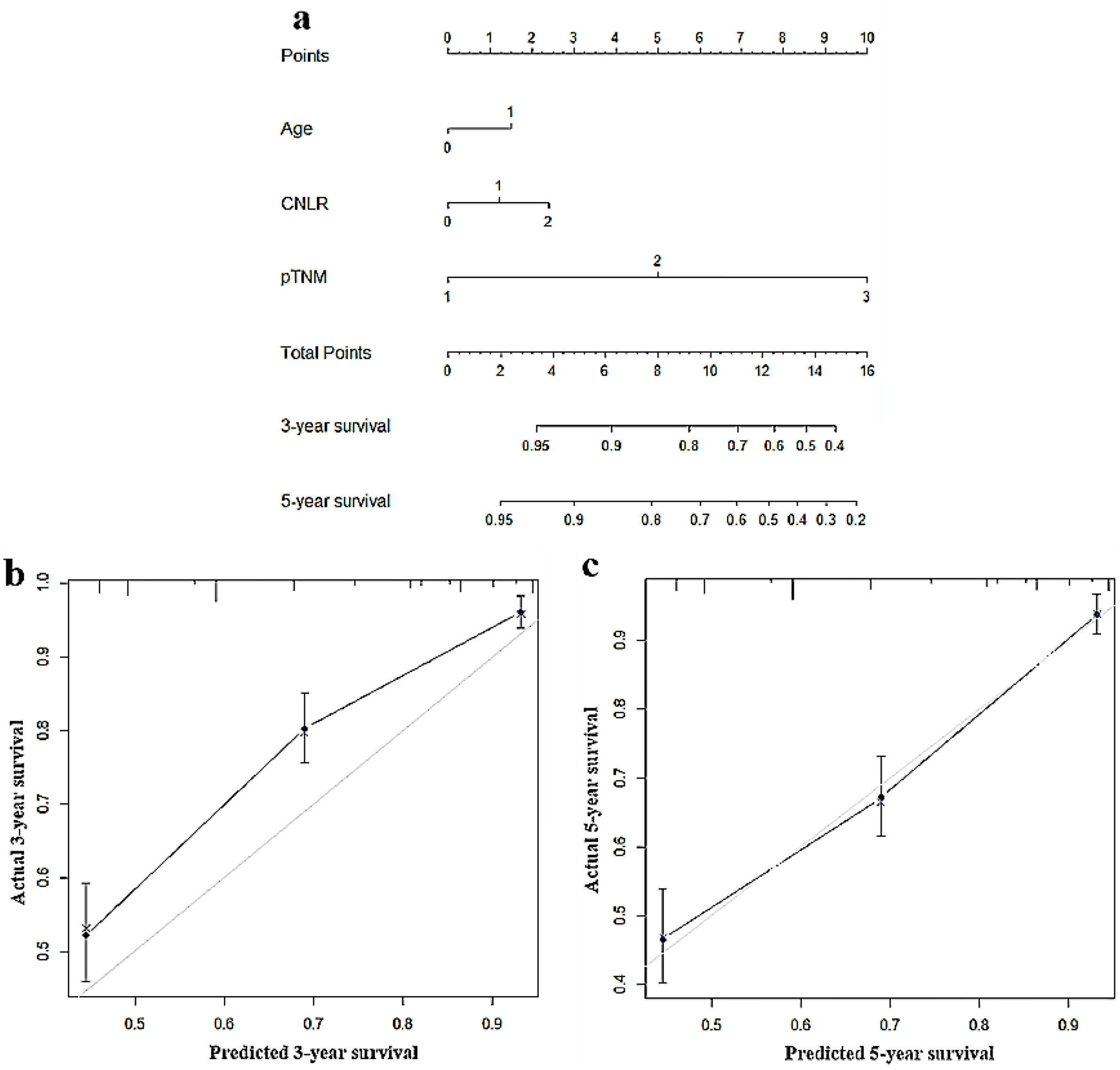

Supplementary Fig. 3 Time-dependent ROC curves for the nomogram, non-CNLR nomogram, and pTNM for the prediction of OS in the validation set. The horizontal axis represents the years after surgery, and the vertical axis represents the estimated area under the ROC curve for survival at the time of interest. Green, blue, and red solid lines represent the estimated AUCs of the nomogram, non-CNLR nomogram, and pTNM, respectively. Broken lines represent the 95% confidence interval of each AUC

Abbreviations: ROC receiver-operating characteristic, *CNLR* combination of carcinoembryonic antigen and neutrophils to lymphocytes ratio, *AUC* area under the curve

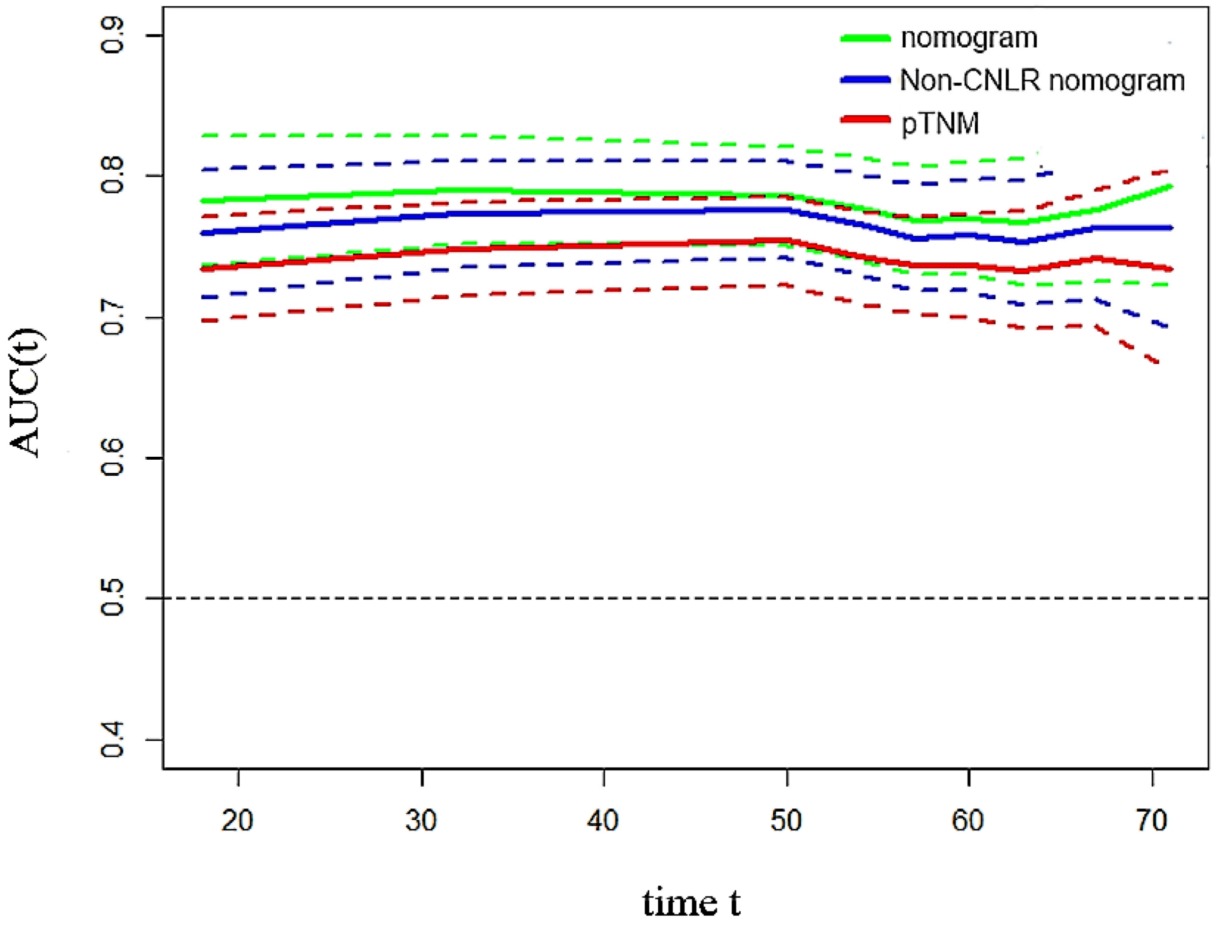

Supplementary Table 1 Clinicopathologic characteristics of the training set and the validation set

Clinicopathological features	Training set<br>All cases = 803	Validation set<br>All cases = 794
**Patient characteristics**		
Age (years, median, IQR)	58 (49–64)	61 (55–68)
Gender		
Female	197 (24.5%)	190 (23.9%)
Male	606 (75.5%)	604 (76.1%)
ASA score		
1	628 (78.2%)	495 (62.3%)
2	146 (18.2%)	267 (33.6%)
3	29 (3.6%)	32 (4.0%)
**Disease characteristics**		
Tumour location		
Upper	194 (24.2%)	191 (24.1%)
Middle	408 (50.8%)	148 (18.6%)
Lower	194 (24.2%)	338 (42.6%)
Mixed	7 (0.9%)	117 (14.7%)
Tumour size (cm, median, IQR)	4.0 (2.5–5.0)	4.0 (2.5–6.0)
Tumour differentiation		
Differentiated	378 (47.1%)	173 (21.8%)
Undifferentiated	425 (52.9%)	621 (78.2%)
Vascular invasion		
Negative	264 (32.9%)	555 (69.9%)
Positive	434 (54.0%)	239 (30.1%)
Unknown	105 (13.1%)	—
pTNM stage		
I	262 (32.6%)	221 (27.8%)
II	210 (26.2%)	194 (24.4%)
III	331 (41.2%)	379 (47.7%)
Adjuvant chemotherapy		
Yes	440 (54.8%)	413 (52.0%)
No	363 (45.2%)	381 (48.0%)
ALB (g/L)	39.3 (36.80–42.00)	40.35 (36.70–43.00)
Hb (g/L)	145.00 (125.00–159.00)	131.00 (115.00–144.00)
CEA (ng/mL, median IQR)	2.13 (1.38–3.56)	2.60 (1.60–4.60)
**SIR markers (median, IQR)**		
LMR	4.44 (2.64–5.83)	4.27 (3.20–5.55)
PLR	116.05 (84.07–194.29)	139.08 (102.49–183.38)
NLR	2.01 (1.45–3.49)	2.11 (1.57–2.88)

ASA American Society of Anesthesiologists, *IQR* interquartile range, *ALB* albumin, *Hb* hemoglobin, *CEA* carcinoembryonic antigen, *SIR* systemic inflammatory response, *LM*R lymphocyte-to-monocyte ratio, *PL*R platelet-to-lymphocyte ratio, *NLR* neutrophil-to-lymphocyte ratio

Supplementary Table 2 The relationship between the CNLR and clinicopathological characteristics in patients undergoing radical gastrectomy in the validation set

Clinicopathological features	CNLR			*p*
	0	1	2	
Case	197 (24.8%)	369 (46.5%)	228 (28.7%)	
Age (years, $\bar{x} \pm$ s)	57.66 ± 10.86	60.53 ± 1064	64.10 ± 9.22	0.045
Gender				<0.001
Female	82 (33.6%)	89 (21.9%)	26 (17.1%)	
Male	162 (66.4%)	318 (78.1%)	126 (82.9%)	
ASA score				0.506
1	133 (67.5%)	222 (60.2%)	140 (61.4%)	
2	58 (29.4%)	130 (35.2%)	79 (34.6%)	
3	6 (3.0%)	17 (4.6%)	9 (3.9%)	
Tumour location				0.001
Upper	32 (16.2%)	84 (22.8%)	75 (32.9%)	
Middle	43 (21.8%)	63 (17.1%)	42 (18.4%)	
Lower	93(47.2%)	172(46.6%)	73(32.0%)	
Mixed	29 (14.7%)	50 (13.6%)	38 (16.7%)	
Tumour size (cm, $\bar{x} \pm$ s)	4.16 ± 2.28	4.30 ± 2.30	5.17 ± 2.84	0.003
Tumour differentiation				0.203
Differentiated	34 (17.3%)	85 (23.0%)	54 (23.7%)	
Undifferentiated	163 (82.7%)	284 (77.0%)	174 (76.3%)	
Vascular invasion				<0.001
Negative	159 (80.7%)	257 (69.6%)	139 (61.0%)	
Positive	38 (19.3%)	112 (30.4%)	89 (39.0%)	
Adjuvant chemotherapy				0.215
No	104 (52.8%)	176 (47.7%)	101 (44.3%)	
Yes	93 (47.2%)	193 (52.3%)	127 (55.7%)	
pTNM stage				<0.001
I	77 (39.1%)	105 (28.5%)	39 (17.1%)	
II	53 (26.9%)	92 (24.9%)	49 (21.5%)	
III	67 (34.0%)	172 (46.6%)	140 (61.4%)	
Postoperative morbidity				0.076
No	164 (83.2%)	305 (82.7%)	173 (75.9%)	
Yes	33 (16.8%)	64 (17.3%)	55 (24.1%)	

CNLR combination of carcinoembryonic antigen and neutrophils to lymphocytes ratio, *ASA* American Society of Anesthesiologists

Supplementary Table 3 The definition of CNLR

Parameters	Group 1	Group 2	Group 3	Group 4
CEA	<3.04	≥3.04	<3.04	≥3.04
CEA score	0	1	0	1
NLR	< 1.86	<1.86	≥1.86	≥1.86
NLR score	0	0	1	1
CNLR score	0	1	1	2

CNLR combination of carcinoembryonic antigen and neutrophils to lymphocytes ratio, *CEA* carcinoembryonic antigen, *NLR* neutrophils to lymphocytes ratio
